# Zinc Modulates Several Transcription-Factor Regulated Pathways in Mouse Skeletal Muscle Cells

**DOI:** 10.3390/molecules25215098

**Published:** 2020-11-03

**Authors:** Parisa Vahidi Ferdowsi, Rachel Ng, John Adulcikas, Sukhwinder Singh Sohal, Stephen Myers

**Affiliations:** College of Health and Medicine, School of Health Sciences, University of Tasmania, Newnham Campus, Launceston 7250, Australia; Parisa.VahidiFerdowsi@utas.edu.au (P.V.F.); rc.ng@utas.edu.au (R.N.); johna6@utas.edu.au (J.A.); sukhwinder.sohal@utas.edu.au (S.S.S.)

**Keywords:** zinc, skeletal muscle, transcription factors, cell signalling, insulin

## Abstract

Zinc is an essential metal ion involved in many biological processes. Studies have shown that zinc can activate several molecules in the insulin signalling pathway and the concomitant uptake of glucose in skeletal muscle cells. However, there is limited information on other potential pathways that zinc can activate in skeletal muscle. Accordingly, this study aimed to identify other zinc-activating pathways in skeletal muscle cells to further delineate the role of this metal ion in cellular processes. Mouse C2C12 skeletal muscle cells were treated with insulin (10 nM), zinc (20 µM), and the zinc chelator TPEN (various concentrations) over 60 min. Western blots were performed for the zinc-activation of pAkt, pErk, and pCreb. A Cignal 45-Reporter Array that targets 45 signalling pathways was utilised to test the ability of zinc to activate pathways that have not yet been described. Zinc and insulin activated pAkt over 60 min as expected. Moreover, the treatment of C2C12 skeletal muscle cells with TPEN reduced the ability of zinc to activate pAkt and pErk. Zinc also activated several associated novel transcription factor pathways including Nrf1/Nrf2, ATF6, CREB, EGR1, STAT1, AP-1, PPAR, and TCF/LEF, and pCREB protein over 120 min of zinc treatment. These studies have shown that zinc’s activity extends beyond that of insulin signalling and plays a role in modulating novel transcription factor activated pathways. Further studies to determine the exact role of zinc in the activation of transcription factor pathways will provide novel insights into this metal ion actions.

## 1. Introduction

Zinc is one of the most important trace elements in biological systems. It is ubiquitous and is responsible for many biological processes [1] including growth and immunity, development [2], nucleic acid and lipid metabolism, apoptosis, and brain function [3]. It is essential for the catalytic activity of over three hundred enzymes and is involved in many aspects of cellular metabolism and cell signalling processes [4,5,6].

Zinc is critical for maintaining cellular zinc concentrations at physiological levels and any deviations in the homeostatic concentrations of zinc can be detrimental for a cell and its processes [7]. Mechanisms that influence dysfunctional zinc signalling are associated with metabolic disease states including cancer [8], cardiovascular disease [9], Alzheimer’s disease [10], and diabetes [11]. Accordingly, zinc movement in cells is tightly controlled by a diverse number of zinc transporters that facilitate zinc flux and therefore maintain cellular zinc homeostasis [2]. Zinc stimulates the activity of growth factors, hormones, and cytokines [12]. Given the diverse number of zinc transporters involved in modulating zinc homeostasis, it is becoming more evident that this metal ion is taking precedence as a leading cell signalling molecule, analogous to calcium signalling [2].

Recent studies have highlighted zinc’s diverse role as a “cellular second messenger” in the modulation of insulin signalling and glucose homeostasis [13]. The mechanisms of zinc action are essential for the synthesis, storage, and secretion of insulin from pancreatic beta-cells [14]. Early studies have demonstrated zinc increases glucose metabolism in isolated rat adipocytes [15,16]. In 3T3-L1 adipocyte cells, zinc treatment increased tyrosine phosphorylation of insulin receptor β subunit and Akt phosphorylation. These actions were concomitant with enhanced glucose transport into 3T3L1 cells, independent of insulin [17]. Similarly, the treatment of human and mouse C2C12 skeletal muscle cells with zinc led to significant phosphorylation of Akt, Erk, Gsk-3β, p38, and tyrosine [15]. Moreover, zinc treatment resulted in significant oxidation of glucose in both cell types [15].

In normal rat L6 myotubes, glucose consumption was enhanced via the zinc activation of Akt phosphorylation, glucose transporter translocation, and Gsk-3β phosphorylation [18,19]. Chelation of cellular zinc with the membrane-permeable N,N,N’,N’-tetrakis(2-pyridylmethyl)ethylenediamine (TPEN) suppressed insulin and IGF-1-stimulated phosphorylation [19]. Zinc can also inhibit protein tyrosine phosphatases that result in enhanced insulin/IGF-1-receptor phosphorylation and the activation of subsequent cell signalling cascades [20]. While these studies on zinc provide crucial information into the complex role of this metal ion in cell signalling pathways, more information is needed regarding other potential molecules and cellular pathways influenced by zinc. Accordingly, this research aimed to establish a more detailed and robust analysis of potential cellular pathways that are activated by zinc. This research is therefore highly significant based on the novel data of zinc-mediated cellular signalling in skeletal muscle cells. These data, therefore, enrich the research field of zinc biology and will contribute to novel findings from other cells, and or, tissue-based systems.

## 2. Results

### 2.1. MTT Assay of Cell Viability in C2C12 Cells under the Treatment of Insulin, Zinc, Sodium Pyrithione, and TPEN at Different Timepoints

**Figure 1:**
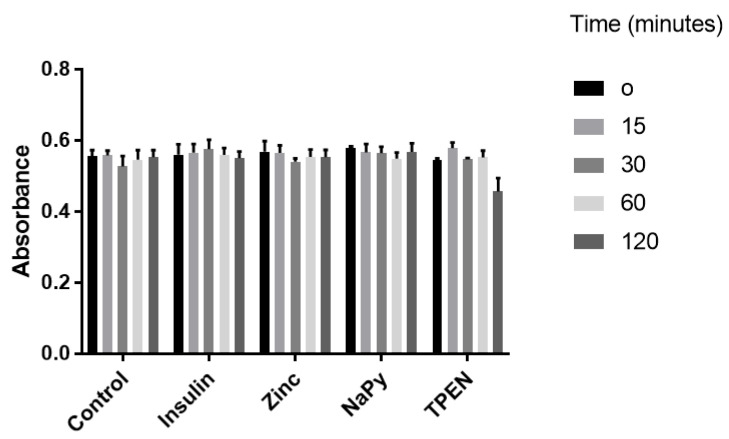
MTT assay of C2C12 cells following treatment with 10 nM of insulin, 20 µM of zinc, 10 µM of NaPy, and 100 µM of TPEN for 0, 15, 30, 60, and 120 min.

C2C12 skeletal muscle cells were utilised to assess cell viability under different treatments. These cell lines have previously been used to assess the zinc-mediated activation of several cell signalling pathways influenced by insulin signalling [15,21,22]. We treated C2C12 cells with 10 nM of insulin, 20 µM of zinc, 10 µM of NaPy, and 100 µM TPEN over 120 min (Figure 1), as previously described [15,21,22,23,24]. It was identified that these concentrations of insulin, zinc, NaPy, and TPEN did not significantly suppress C2C12 cell viability (Figure 1), except for the 100 µM of TPEN at the 120-min time point. Accordingly, we used 60 min of TPEN treatment and 30 min for insulin, zinc, and sodium pyrithione for subsequent experiments (Figure 1).

### 2.2. Effect of Zinc and Insulin on Insulin-Dependent Cell Signalling Molecule Akt in Mouse Skeletal Muscle Cells

**Figure 2:**
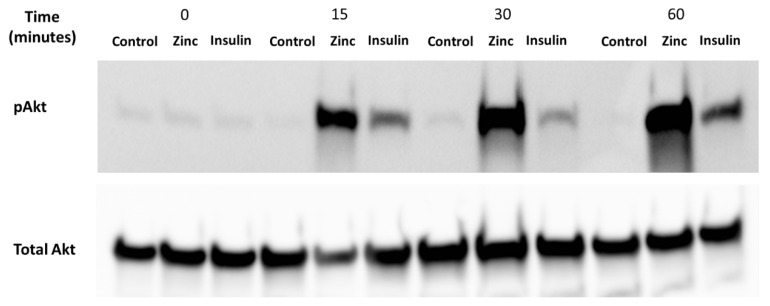
Analysis of pAkt in C2C12 mouse skeletal muscle cells treated with control (DMSO), zinc, NaPy, and insulin over 60 min. Time is shown from 0, 15, 30, and 60 min and total Akt was used as an internal loading control. The levels of pAkt were normalised to total Akt.

It is well-established that zinc can activate Akt, a protein that is phosphorylated under the presence of insulin and zinc [25]. Accordingly, it was important to determine whether the C2C12 cells were viable and responded to these treatments before assaying for zinc using the Cignal 45 Reporter Array. C2C12 mouse skeletal muscle cells were treated with 10 nM insulin, 20 µM of zinc, and 10 µM of NaPy over a time period of 0, 15, 30, 60 min (Figure 2). We observed a significant increase in the phosphorylation of Akt by insulin and zinc over a 60-min time point (Figure 2).

### 2.3. Effect of Zinc Chelator, TPEN, on Phosphorylation of Akt and Erk Following Treatment of Zinc and Sodium Pyrithione in C2C12 Mouse Skeletal Muscle Cells

**Figure 3:**
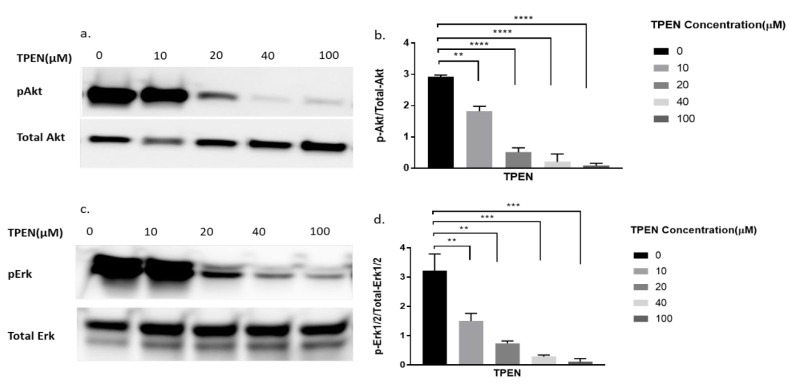
Western blot result of pAkt and pErk in C2C12 cells treated with increasing concentrations of TPEN. (**a**,**c**). Cells were treated with increasing concentrations of TPEN (0, 10, 20, 40, 100 µM) for 60 min followed by 30 min of 20 µM zinc and 10 µM NaPy treatment before harvesting total protein. Phosphorylated Akt and Erk were immunoprobed by Western blotting. Total Akt and total Erk were used as internal controls. Three independent Western blots on three independent treatments were performed. (**b**,**d**). Densitometry graphs for pAkt and pErk, respectively, from three independent data Western blots ** = *p* < 0.01, *** = *p* < 0.001, **** = *p* < 0.0001.

To further delineate if zinc was indeed having the effect observed in Figure 2, we decided to perform a more robust experiment. We utilised TPEN, a well-known membrane-permeable zinc chelator [26] to test for diminished cellular zinc on the phosphorylation of Akt and Erk. Previous studies have identified that zinc activates pAkt and pErk in C2C12 cells [15,22]. Accordingly, the phosphorylation status of Akt and Erk in C2C12 cells was assessed upon increasing concentrations of TPEN (0, 10, 20, 40, and 100 µM) for one hour before treatment with 20µM of zinc and 10µM of NaPy for 30 min. The phosphorylation of Akt and Erk diminished as concentrations of TPEN increased with approximately 40µM of TPEN required to chelate zinc and inhibit Akt and Erk phosphorylation (Figure 3a–d).

### 2.4. Effect of Zinc Chelator, TPEN, on the Phosphorylation of Akt, and Erk under the Treatment of Insulin in C2C12 Mouse Skeletal Muscle Cells

**Figure 4:**
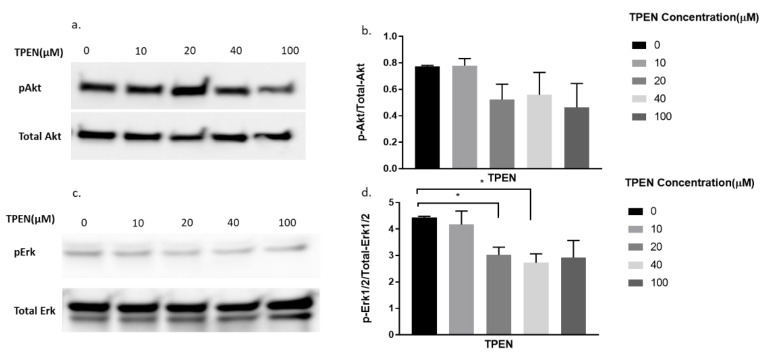
Western blot result of pAkt and pErk in C2C12 cells treated with increasing concentrations of TPEN. (**a**,**c**). Cells were treated with increasing concentrations of TPEN (0, 10, 20, 40, 100 µM) for 60 min followed by 30 min of 10 nM insulin treatment before harvesting total protein. Phosphorylated Akt and Erk were immunoprobed by Western blotting. Total Akt and Total Erk were used as internal controls. Three independent Western blots on three independent treatments were performed. (**b**,**d**). Densitometry graphs for pAkt and pErk, respectively, from three independent Western blots * = *p* < 0.05.

Since TPEN is a zinc chelator, we wanted to test the effect of insulin, independent of zinc, on proteins involved in the insulin signalling pathway under the presence of TPEN. Similarly, increasing concentrations of TPEN were administered to cells for 1 h, followed by 10 nm of insulin treatment for 30 min. As expected, the phosphorylation of Akt did not change under increasing concentrations of TPEN (0, 10, 20, 40, 100 µM) (Figure 4a,b). However, a significant reduction in pErk was observed in the TPEN-treated (20 and 40 µM) C2C12 cells (Figure 4c,d).

### 2.5. Effect of Zinc Chelator, TPEN, on the Phosphorylation of Akt and Erk under the Combined Treatment of Zinc, Sodium Pyrithione, and Insulin in C2C12 Mouse Skeletal Muscle Cells

**Figure 5:**
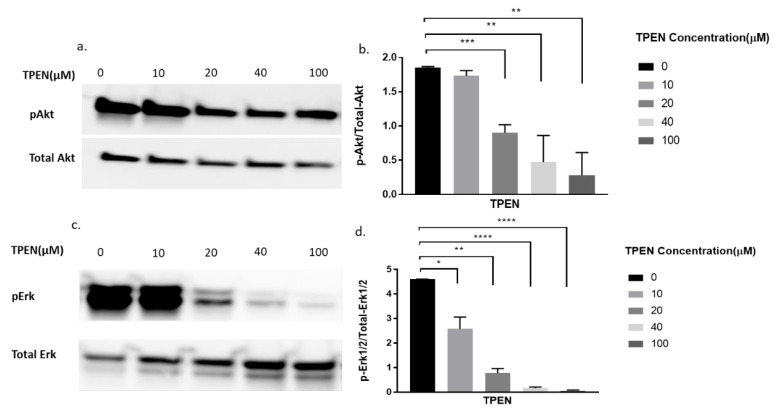
Western blot result of pAkt and pErk expression in C2C12 cells treated with increasing concentrations of TPEN. (**a**,**c**). Cells were treated with increasing concentrations of TPEN (0, 10, 20, 40, 100 µM) for 60 min followed by 30 min of 20 µM zinc,10 µM NaPy and 10nM insulin treatment before harvesting total protein. Phosphorylated Akt and Erk were immunoprobed by Western blotting. Total Akt and Total Erk were used as internal controls. Three independent Western blots on three independent treatments were performed. (**b**,**d**). Densitometry graphs for pAkt and pErk, respectively, from three independent data Western blots * = *p* < 0.05, ** = *p* < 0.01, *** = *p* < 0.001, **** = *p* < 0.0001.

From the above experiments, it was clear that zinc was chelated by TPEN and insulin was unaffected by TPEN. We combined both treatments to demonstrate any augmented effects of zinc and insulin under TPEN treatment. Accordingly, cells were treated with increasing concentrations of TPEN for one hour followed by zinc, NaPy, and insulin treatment for 30 min. There was a significant decrease in Akt phosphorylation at 20, 40, and 100 µM of TPEN treatment (Figure 5a,b). Erk phosphorylation also showed a significant decrease in these concentrations of TPEN treatment (Figure 5c,d).

### 2.6. Zinc Treatment of a Cignal Finder Reporter Array for the Analysis of Forty-Five Unique Signalling Pathways

Zinc activates well-established proteins pAkt and pErk which are implicated in insulin signalling, and involved in glucose homeostasis in skeletal muscle [15]. While there are many studies on zinc and the activation of key insulin signalling molecules, there are limited studies on other potential activation pathways. Accordingly, we used a Cignal Finder Reporter Array (Qiagen, Australia) containing forty-five unique transcription factor binding sites to analyse the effect of zinc-activation of these DNA response elements and the subsequent analysis of the potential regulation of up-and/or down-stream pathways (Table 1). Following the treatment of C2C12 cells with 20 µM zinc for 16 h, the changes in DNA response element-luciferase activation were assessed. We observed that zinc activated several transcriptional DNA response elements including Nrf1/Nrf2, ATF6, CREB, EGR1, STAT1, AP-1, PPAR, and TCF/LEF (Table 1).

Changes in the activation of the transcription factor are presented as (1) pathway name (transcription factor), (2) fold up- or down-regulation, and (3) the p-value. Results in bold are considered statistically significant.

### 2.7. Validation of cAMP Response Element-Binding Protein (CREB) in Response to Zinc

**Figure 6:**
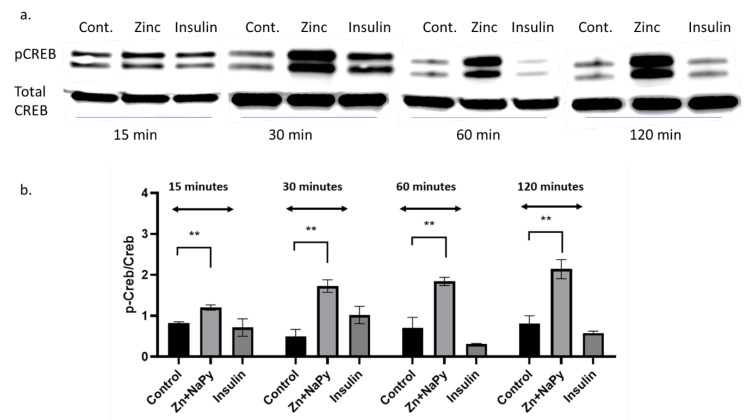
Western blot and densitometry of pCREB expression in C2C12 cells treated with zinc and insulin over 120 min. (**a**). Western blot of pCREB and total CREB treated with 20 µM zinc or 10 nM of insulin over 15, 30, 60, and 120 min. (**b**). Densitometry plot of pCreb/Creb from the Western blot data (**a**) from three independent experiments, ** = *p* < 0.01. Control = no treatment. Zn + NaPy = zinc plus sodium pyrithione.

The zinc-mediated changes observed on various signal reporter pathways encouraged us to validate the CREB response observed in Table 1. Because zinc has signalling properties [2], we sought to validate how zinc might activate another well-known signalling molecule, CREB. We identified that 20 μM of zinc activated the phosphorylation of CREB in C2C12 mouse skeletal muscle cells over 120 min of incubation with zinc (Figure 6a,b).

### 2.8. Reactome Analysis of Significant Transcription Factors and Subsequent Pathway Analysis

Reactome analysis tool was used for visualisation, interpretation, and analysis of transcription factor pathways. Given the transcription factors that were significantly upregulated upon zinc treatment (Table 1), we sought to investigate these pathways further (Table 2). Following analysis of the response elements from each of the transcription factors that were significantly activated by zinc (Table 1), a detailed pathway analysis was performed. Table 2 shows the results of the Reactome analysis where the ”X” represents the various pathways in which each of the transcription factors is subsequently implicated.

## 3. Discussion

In this study, we explored novel cell signalling pathways that zinc could potentially activate, other than those previously reported for insulin signalling.

Initially, it was essential to determine the viability and response of the C2C12 cells to well-established treatments and subsequent protein outputs [22,27]. Accordingly, we set out to demonstrate the properties of zinc through well-established experimentation and to determine the response status of the C2C12 skeletal muscle cells. Protein kinase B, commonly known as Akt is a protein that plays a significant role in insulin signalling where increased Akt phosphorylation is known to be accompanied by a concomitant increase in glucose transport in skeletal muscles [28]. We observed that C2C12 cells treated with insulin or zinc increased Akt phosphorylation within 15 min of treatment and this is supported by previously published data in skeletal muscle cells [15,21,22,29]. It appears that the activation of pAkt by zinc is more robust compared to insulin treatment. It is not clear why the pAkt response to zinc is augmented. It has been shown that C2C12 cells do not express sufficient levels of Glut4 protein and their insulin response is somewhat blunted [30]. Perhaps these cells are more sensitive to zinc and this might explain the observed reduction of insulin-mediated pAkt when compared with zinc. However, our previous studies with C2C12 cells have found this cell line expresses Glut4 and is sensitive to insulin-mediated glucose uptake [15,21,22].

To further delineate that zinc indeed was responsible for the phosphorylation of Akt we utilised the zinc chelator, TPEN. TPEN is a lipid-soluble zinc metal chelator [26] that depletes zinc in cells [31] and therefore inhibits the effects of zinc. Proteins such as Akt and components of the mitogen-activated protein kinase/extracellular signal-regulated protein kinase (MAPK/ERK) pathways modulate the responsiveness of cellular insulin [32]. Hence the phosphorylation status of Akt and ERK1/2 were compared in the presence of TPEN, zinc, insulin, and a combination of zinc and insulin.

Following treatment with zinc and increasing concentrations of TPEN, the phosphorylation status of Akt and Erk1/2 was significantly diminished. These data suggest that intracellular zinc can phosphorylate Akt and Erk1/2. C2C12 cells treated with insulin and increasing concentrations of TPEN showed no observable changes in the phosphorylation status of Akt or Erk1/2. This suggests that TPEN-mediated zinc chelation does not affect insulin-mediated activation of these downstream molecules and is further supported by previous studies where TPEN-mediated zinc chelation did not affect insulin gene expression in INS-1E cells [33]. However, the combined treatment of zinc and insulin treatment under increasing TPEN concentrations showed an observable and significant decrease in the phosphorylation status of Akt and Erk1/2. This is most likely due to TPEN’s chelating effect on zinc and the residual phosphorylation of Akt and Erk1/2 observed could be solely due to insulin. Similarly, this might also suggest that insulin is dependent on zinc for the phosphorylation of Akt and Erk1/2. A recent study found that the chemically-induced inhibition of insulin receptor tyrosine kinase activity in C2C12 cells abolished the ability of insulin and zinc to phosphorylate Akt and therefore suggests that the insulin-mediated pAkt is potentially dependent on zinc action [15]. These data are also supported by a previous study in rat L6 skeletal muscle cells where insulin and zinc independently activated downstream pathways including Akt, Glut4, and glucose consumption, however, the co-incubation of insulin and zinc failed to activate these same molecules and the glucose response [18].

Following the responsive effect of zinc on activating key, well-established downstream molecules in C2C12 cells, we set out to determine the effect of zinc on multiple cellular pathways that are induced by transcription factors. Accordingly, a Cignal Reporter Array containing 45 unique transcription factor binding sites was utilised. Following zinc treatment, significant changes in several transcription factors including Nrf1/Nrf2, ATF6, CREB, EGR1, STAT1, AP1, PPAR, and TCF/LEF were identified and their subsequent pathways were analysed bioinformatically. It is not clear whether zinc directly interacts with these transcription factors, or indirectly targeting other proteins or signalling molecules that could activate the above-mentioned transcription factors. However, from the above transcription factors, some extrapolation could provide information that will warrant further investigation.

Bioinformatics analysis of Nrf1/Nrf2, ATF6, CREB, EGR1, STAT1, AP1, PPAR and TCF/LEF, revealed these transcription factors to be involved in multiple pathways implicated in the immune system, signal transduction, gene expression, metabolism of proteins, programmed cell death, the circadian clock, the cell cycle, cell–cell communication, DNA repair, transport of small molecules, and cellular responses to stress (Table 2) [34,35,36,37,38,39,40,41,42,43,44,45,46,47,48,49,50,51,52,53,54,55,56,57,58,59,60].

Nrf1 and Nrf2 are transcription factors involved in the antioxidant response [61]. Zinc is known to act as an antioxidant by affecting the expression of glutathione via the nuclear factor erythroid 2 (NFE2)-related factor 2 (Nrf2)-dependent pathways [62,63]. The overexpression of Nrf1 in mouse skeletal muscle resulted in increased expression of mitochondrial proteins cytochrome C, ALA synthase, and ubiquinol cytochrome c oxidoreductase, and this was concomitant with an increase in insulin-stimulated glucose transport [64].

ATF6 is associated with an adaptive response to endoplasmic reticulum (ER) stress and is concomitant with the induction of insulin resistance in skeletal muscle [65]. A study has demonstrated that zinc treatment in the livers of ER-stress induced OVE26 transgenic type 1 diabetic mice showed improvements in ER stress injury concomitant with reduced ATF6 expression [66]. In another study, the zinc transporter Zip14 mediated hepatic zinc uptake and played an important role in suppressing ER stress-induced apoptosis [67]. It is suggested that during ER stress, ATF6 transcriptionally upregulated Zip14 to enhance zinc concentration intracellularly to inhibit PTP1B activity and contribute to overcoming ER stress [67]. Our studies showed that zinc could activate ATF6. Whether zinc activates ATF6 directly or was the consequence of the treatment of zinc activating the ER-stress pathway is not clear. It is unlikely that the 20 µM zinc treatment in these studies activated the ER stress response as this concentration (and much higher) and has been used in several studies as the baseline concentration of zinc for cell signalling studies [15,21,23,68].

CREB (cAMP response element-binding protein) is activated in response to a wide range of physiological stimulus that leads to targeted gene transcription and cellular responses [69]. Our data demonstrated that zinc treatment significantly increases the phosphorylation and activation of CREB over 120-min incubation. This confirms that zinc stimulates various cellular pathways by activation of transcription factors including CREB. While the literature on zinc activation of CREB is limited, there are data on the role of zinc transporter proteins in the modulation of CREB. Zinc transporter (Zip14) knock out mice have impaired growth and skeletal muscle development due to impaired CREB activation [70]. Another zinc transporter, Zip4, is reported to increase interleukin 6 transcription through CREB, resulting in cell proliferation and tumour progression in pancreatic cancer cells [71]. While it is possible that zinc treatment of C2C12 cells resulted in the upregulation of Zip 4 and subsequent activation of the CREB pathway, regulated Zip4 expression by zinc availability is poorly understood [72]. Similarly, overexpression of the zinc transporter ZIP7 in MCF-7 breast cancer cell lines led to the activation of pCREB [73].

Egr1 is involved in the early growth response gene-1 involved in proliferation, differentiation and cell death pathways [74]. A study demonstrated how zinc could induce Egr1 expression in mouse cortical cultures via the activation of Erk1/2 [75]. Similarly, zinc depletion in HepG2 cells reduced Erg1 expression, and this was associated with decreased ApoA1 gene expression [76]. In C2C12 cells, Egr1 mRNA was increased during cellular differentiation [77], however, the role of zinc and the activation of Egr1 in this cell line is not clear.

STAT1 is part of the JAK1-STAT1-STAT3 pathway that has dual roles in proliferating myoblasts including proliferation, and differentiation [78]. A study has shown that zinc chelation decreased STAT1 upregulation in macrophages and this may be relevant to elderly people suffering from a higher risk of bacterial infection because of zinc deficiency. However, as the experiment was based on one isolated cell type, it does not represent the complexity of zinc deficiency in the human population [79]. In the central nervous system (CNS), zinc deficiency has been demonstrated to disrupt STAT1 signalling in the development of the foetal CNS. Zinc deficiency also results in oxidative stress in embryonic and brain cells through compromised tyrosine phosphorylation of STAT1 [80]. However, the regulation of zinc in STAT1 pathway in skeletal muscle cells remains poorly understood.

Activator protein -1 (AP-1) is involved in differentiation, proliferation, and apoptotic cell death. A study showed that in zinc deficiency, AP-1 signalling was altered in primary T cells of young and elderly healthy individuals. Supplementation of zinc restored AP-1 activation in the T-cells of the young but not in the elderly. This suggests the complex mechanics involved in zinc deficiency [81].

Peroxisome proliferator-activated receptors (PPAR) possess potent anti-inflammatory signalling properties [82]. The DNA binding domains of PPAR have two sets of zinc fingers and zinc is an important constituent of the DNA binding domains in PPAR, hence zinc deficiency could result in the impairment of PPAR signalling during endothelial cell activation [83].

T-cell factor/lymphoid enhancer factor (TCF/LEF) transcription factors appear to be involved in the immune system, signal transduction, and programmed cell death. To our knowledge, no data exists showing how zinc could cause TCF/LEF activation. However, there are reports of TCF containing a zinc-finger domain in the cysteine clamp (C-clamp) [84] and the C-clamp is a zinc-binding domain [85]. Similarly, another study showed how zinc fingers could be involved in controlling gene expression in response to zinc status [86]. Hence it could be possible that the upregulation of TCF/LEF transcription factors was due to zinc finger’s response to zinc status.

## 4. Materials and Methods

### 4.1. Cell Culture

Mouse C2C12 cells were a generous gift from Professor Steve Rattigan, Menzies Institute for Medical Research, Hobart, Australia. Other C2C12 cells were purchased from Sigma-Aldrich, Australia (Catalog number, 91031101). C2C12 cells were grown and cultured in Dulbecco’s Modified Eagle Medium (DMEM) (Thermo Fisher, Victoria, Australia) that contained 10% foetal calf serum (FCS) and 100 U/mL penicillin/streptomycin (Thermo Fisher) and were maintained at 37 °C and 5% CO_2_ in a humidified atmosphere. Prior to the different cell treatments, cells were exposed to serum-free conditions for three hours. The cells were then exposed to different treatments as outlined below.

### 4.2. Cell Viability Assay [1-(4,5-Dimethylthiazol-2-yl)-3,5-diphenylformazan] Assay

Cell viability was measured using the MTT (3-(4,5)-dimethylthiahiazo(−z-y1)-3,5-diphenytetrazoliumromid) assay according to manufacturer’s instructions (Thermo Fisher). Briefly, C2C12 skeletal muscle cells were seeded into a 96-well plate and treated with insulin (10 nM), zinc (20 µM), sodium pyrithione (10 µM), and TPEN (100 µM) at various timepoints (as described in Results). Then, 10 µL of 12 mM MTT solution was added to each well and incubated at 37 °C for 2 h. After 2 h, all but 25 µL of the medium from wells was removed. To dissolve the formazan produced, 50 µL of dimethyl sulfoxide (DMSO) was added into each well and incubated at 37 °C for 10 min. Samples were measured at 540 nm using a microplate reader (TECAN infinite M200 PRO, Männedorf, Switzerland).

### 4.3. Protein Extraction

Whole-cell lysates were prepared in RIPA lysis buffer in the presence of protease and protein phosphatase inhibitors (Thermo Fisher) as previously described [15]. Lysates were vortexed every 10 min for 1 h at 4 °C and centrifuged at 15,000× *g* rpm. Protein concentrations of the supernatants were determined using the BCA assay kit as per the manufacturer’s instructions (Thermo Fisher).

### 4.4. Western Blot Analysis

Western blots were performed as previously described [15]. Briefly, total cellular protein (20 µg) was heated to 95 °C and electrophoresed on 4–15% SDS polyacrylamide gels (BioRad, NSW, Australia). Immunoblotting was performed using a semi-dry electrophoresis blotting system (BioRad, Trans-Blot^®^ Turbo™ System). The membranes were blocked for 2 h in TBST (50 mmol/L Tris-Cl, pH 7.6, 150 mmol/L NaCl and 0.1% Tween 20) containing casein and incubated with the appropriate primary antibody overnight at 4 °C. Primary antibodies used were phosphorylated Akt (1:5000; Cell Signalling, Catalogue number 4058), phosphorylated CREB (1:2000; Cell Signalling, Catalogue number 9198), and phosphorylated ERK (1:2500; Cell Signalling, Catalogue Number 8544). Membranes were washed four times with TBST and HRP-linked secondary Anti-Rabbit antibodies (Cell Signalling, Catalogue Number 7074) were incubated with the membranes for 1 h at room temperature. Membranes were then stripped with Restore Plus Western Blot Stripping Buffer (Thermo Fisher) and probed again for the related housekeeping gene (see Results Figure 3, Figure 4 and Figure 5). All phosphor-immunoreactive species, Akt, CREB, and ERK were normalised against total Akt (1:5000, Cell Signalling, Catalogue Number 9272), total CREB (1:5000, Cell Signalling, Catalogue Number 9197), and ERK (1:5000, Cell Signalling, Catalogue Number 9102). Proteins were visualised with Super Signal West Femto Kit (Thermo Fisher) and imaged using an Odyssey infrared imaging system (LiCor, Millennium Science, Victoria, Australia). Relative band density was quantified using Image J software (https://imagej.nih.gov/ij/). Representative immunoblots are provided from three independent Western blots.

### 4.5. Cignal Finder 45-Pathway Reporter Array

Cignal 45-Pathway Reporter Arrays (Qiagen, Catalogue Number C82DB0D7-3D10-4B1C-A358-411558D2DE01) were utilised to simultaneously assess 45 different dual-luciferase reporter signalling pathways (see Results Table for details). The assay was performed as described by the manufacturers (Qiagen, Cignal Reporter Array Handbook; January 2011). Briefly, C2C12 cells were seeded into wells of the Cignal Finder 96-well Reporter Array and the pathway reporters were introduced into the cells via reverse transfection according to manufacturer’s instructions. Briefly, reporter DNA constructs resident in each well were resuspended with 50 µL of Opti-MEM and 50 µL of diluted Attractene Transfection Reagent. Cells were suspended in Opti-MEM supplemented with 5% of foetal bovine serum at a density of 8 × 10^5^ cells/mL. Then, 50 µL of cell suspension with approximately 40,000 cells was added into each well, mixed with DNA construct in the plate and transfection reagent. The cells were incubated for 16 h at 5% CO_2_ and 37 °C. Following transfection, the medium was replaced with 75 µL of Opti-MEM supplemented with 0.5% of foetal bovine serum, 100 U/mL Penicillin and 100 µg/mL Streptomycin. Twenty-four-hour post-transfection, cells were treated with 20 µM of zinc and incubated for 16 h. Analysis of 45 signalling pathways in the cells was then carried out using the Dual-Glo Luciferase Assay System from Promega as per manufacturer’s instructions (Promega, Catalogue number E1910). Dual Renilla/Luciferase was measured with the Luciferase assay using a microplate reader (TECAN infinite M200 PRO, Männedorf, Switzerland).

### 4.6. Reactome Pathway Database

Reactome is an open-sourced, bioinformatics database for the visualisation, interpretation, and analysis of molecular pathways [87]. The analytic tools enable users to visualise protein–protein interactions and subsequent down- and/or up-stream pathways [86]. Accordingly, the transcription factor pathways significantly activated by zinc in the Cignal Finder 45-Pathway Reporter Array were analysed through the pathway browser tool function [88].

### 4.7. Statistics

Results are presented as means ± SDs. Statistical analysis for the Western blots was performed using Student’s t-test (Prism GraphPad, version 8, San Diego, CA, USA) and normalised to the zero control. Statistical analysis for the Cignal Reporter assay was performed on eight independent plates (4 control + 4 zinc-treatment) and the relative light units of luciferase were normalised to renilla. A Student’s t-test was performed to compare the ratios of luciferase/renilla in both controls versus zinc treatments. *p* < 0.05 was considered significant.

## 5. Conclusions

Zinc is known to activate key signalling molecules in the insulin signalling pathway (e.g., pAkt and pErk1/2). TPEN treatment resulted in the diminished activation of zinc-mediated Akt and Erk1/2 phosphorylation in C2C12 skeletal muscle cells and confirms a zinc-mediated action on these molecules. Our study also delineated several other pathways and transcription factors that zinc could potentially activate in skeletal muscle cells. These include Nrf1/Nrf2, ATF6, CREB, EGR1, STAT1, AP-1, PPAR, and TCF/LEF transcription factors. These transcription factors have several roles in key cellular processes including signal transduction, gene expression, metabolism, the cell cycle, the immune system, and circadian rhythm and therefore warrants further investigation in skeletal muscle and other cells and/or tissue systems. Zinc has been implicated in many disease processes including cancer, diabetes, obesity, and heart disease. These studies presented here could provide insights into zinc-mediated action in pathways associated with these diseases.

## Figures and Tables

**Table 1:** Cignal 45-Pathway Reporter Array (Qiagen).

Pathway (Transcription Factor)	Fold Up- or Down-regulation	*p*-Value
Amino acid deprivation (ATF2/3/4)	1.28	0.460243
Androgen (AR)	1.54	0.246447
Antioxidant response (Nrf1/Nrf2)	1.84	0.03274733
ATF6	2.11	0.0330452
C/EBP	1.81	0.0537373
cAMP/PKA (CREB)	2.13	0.00806593
Cell cycle (E2F)	1.72	0.251203
DNA damage (p53)	2.00	0.0647255
EGR1	1.66	0.0320945
ER stress (CBF/NF-Y/YY1)	1.88	0.260005
Estrogen (ER)	1.40	0.260204
GATA	2.10	0.382748
Glucocorticoid (GR)	2.65	0.136378
Heat shock (HSF-1)	−0.53	0.434913
Heavy Metal (MTF-1)	1.42	0.419621
Hedgehog (Gli)	2.00	0.196115
HNF4	1.06	0.784660
Hypoxia (HIF-1α)	1.07	0.842879
Interferon regulation (IRF1)	2.10	0.10849925
Type I interferon (STAT1/STAT2)	−0.75	0.403768
Interferon gamma (STAT1)	2.20	0.00816463
KLF4	2.08	0.162073
Liver X (LXR)	−0.79	0.553215
MAPK/Erk (SRF/Elk-1)	−0.96	0.901511
MAP/JNK (AP-1)	2.50	0.0200968
MEF2	2.90	0.198518
Myc (c-Myc)	1.65	0.214488
Nanog	1.83	0.408862
Notch (RBP-Jk)	1.41	0.441760
NFκβ	1.05	0.880450
Oct4	2.41	0.266464
Pax6	1.45	0.214952
PI3K/Akt (FOXO)	1.38	0.440598
PKC/Ca^2+^ (NFAT)	1.34	0.646347
PPAR	1.75	0.0592518
Progesterone (PR)	1.99	0.237715
Retinoic acid (RAR)	1.86	0.132229
Retinoid X (RXR)	1.37	0.215849
SOX2	1.39	0.580026
SP1	−0.98	0.964703
STAT3	2.35	0.0423655
TGF-β (Smad2/3/4)	1.98	0.383393
Vitamin D (VDR)	2.96	0.249077
Wnt (TCF/LEF)	2.28	0.0272812
Xenobiotic (AhR)	3.85	0.3272905

**Table 2:** Transcription factor pathway analysis. Each transcription factor that was significantly regulated by zinc is mapped to the various transcription factor pathways.

Transcription Factor Pathway	(Nrf1/Nrf2)	cAMP/PKA (CREB)	MAP/JNK (AP-1)	ATF6	EGR1	STAT1	PPAR	TCF/LEF
Immune System		X	X		X	X	X	X
Signal Transduction	X				X	X	X	X
Gene Expression (Transcription)	X	X				X	X	
Metabolism of Proteins	X			X	X		X	
Programmed Cell Death					X	X		X
Developmental Biology						X	X	
Organelle Biogenesis and Maintenance	X						X	
Circadian Clock	X						X	
Metabolism	X					X		
Cell Cycle	X					X		
Cell–cell Communication								
DNA Repair		X						
Transport of Small Molecules				X				
Generic Transcription Pathway					X			
Cellular Responses to Stress					X			
